# Table Extraction with Table Data Using VGG-19 Deep Learning Model

**DOI:** 10.3390/s25010203

**Published:** 2025-01-01

**Authors:** Muhammad Zahid Iqbal, Nitish Garg, Saad Bin Ahmed

**Affiliations:** Faculty of Science and Environmental Studies, Department of Computer Science, Lakehead University, Thunder Bay, ON P7B 5E1, Canada; miqbal7@lakeheadu.ca (M.Z.I.); ngarg2@lakeheadu.ca (N.G.)

**Keywords:** table extraction model, information extraction, convolutional neural network, deep neural network

## Abstract

In recent years, significant progress has been achieved in understanding and processing tabular data. However, existing approaches often rely on task-specific features and model architectures, posing challenges in accurately extracting table structures amidst diverse layouts, styles, and noise contamination. This study introduces a comprehensive deep learning methodology that is tailored for the precise identification and extraction of rows and columns from document images that contain tables. The proposed model employs table detection and structure recognition to delineate table and column areas, followed by semantic rule-based approaches for row extraction within tabular sub-regions. The evaluation was performed on the publicly available Marmot data table datasets and demonstrates state-of-the-art performance. Additionally, transfer learning using VGG-19 is employed for fine-tuning the model, enhancing its capability further. Furthermore, this project fills a void in the Marmot dataset by providing it with extra annotations for table structure, expanding its scope to encompass column detection in addition to table identification.

## 1. Introduction

Relational tables are abundant sources of valuable information. Each of these tables is characterized by labeled and typed columns, effectively functioning as a structured database. Over the past decade, the enormous amounts of data have been represented through tables that represent summarized information. These tables are not only abundant but also rich and highly useful, prompting significant advancements in the fields of table interpretation and augmentation in recent years. The tables include various types of text, including research articles, data analyses, newspapers, periodicals, invoices, and financial documents. Some times tables are considered as a prime source of displaying important data points for numerous items, facilitating analysis and interpretation.

Researchers have reported considerable attention to enhance the table recognition algorithms over the past decades, recognizing the critical importance of tables in various domains [1]. This collaborated effort has led to significant advancements in the field, enabling more accurate and efficient interpretation of tabular data across a wide range of applications [2]. The major challenge with tables is their designs, which represent in different forms, making it hard for traditional methods to understand them properly. These methods usually look for visual clues like grid lines, spacing between columns, the type of information in each cell, and the how cells are related to each other, including when they overlap or if certain areas are highlighted with colors. While these techniques might work fine for certain types of tables or in certain situations, they not suitable for all kinds of data representation.

Heuristic methods and traditional models often rely on pre-defined rules or basic algorithms to detect and extract tables [2]. These methods work well for simple layouts but struggle when dealing with complex or unusual table designs, such as nested tables or uneven columns. They also have difficulty handling noisy environments, like blurry scans, faded text, or overlapping elements, which are common in real-world documents. Because these methods lack the ability to adapt to variations, their performance is often inconsistent and unreliable in such scenarios.

Our model is designed to overcome these challenges with advanced features. By using VGG-19, a deep learning model, it can extract detailed patterns and features from images, making it capable of recognizing tables in complex and noisy layouts. Additionally, the proposed model integrates semantic information, which helps to understand the structure and meaning of table elements, such as rows and columns. This combination of robust feature extraction and semantic understanding makes our model more accurate and reliable, even in enviroments with challenging documents. VGG-19 is a kind of convolutional neural network (CNN) that is exceptionally well-suited for table extraction tasks due to its inherent capabilities in learning hierarchical features from input data [3,4]. The tables within documents often exhibit complex structures, including various elements such as lines, borders, text regions, and cells. CNNs can automatically learn these intricate features from the input images, enabling them to effectively identify and extract tables [5]. Furthermore, the spatial hierarchy present in tables, with rows and columns forming distinct patterns, aligns naturally with the hierarchical processing of information in CNNs [1]. Through multiple layers of convolutional and pooling operations, CNNs can capture patterns at different spatial scales, facilitating the detection of table elements, regardless of their size or position within the document. Another advantage of CNNs is their translation-invariance property, which allows them to detect features regardless of their location in the input image. This characteristic is particularly beneficial for table extraction, where tables can appear in various positions within documents. In addition, CNNs manipulate shared parameters across different parts of the input data, enabling them to efficiently capture patterns and generalize well to unseen data. In this paper, we proposed a model named Table Extraction Model (TEM), which is a novel deep learning model that influences the natural interaction between table detection and table structure recognition tasks. The proposed model incorporates a pre-trained base network that incorporates VGG-19 characteristics, which are subsequently fine-tuned to improve overall performance.

This research proposes an idea of combining machine learning algorithms with advanced image processing techniques, so that we can accurately identify and outline tables in document images, leading to a significant improvement. Additionally, we ha ve developed a specialized algorithm to minimize false positives when identifying table elements, which should significantly improve the precision of our table extraction system. T-Recs was one of the early successful attempts to tackle table extraction [2,3,4]. T-Recs works by using word bounding boxes as input, organizing them into rows and columns based on their vertical and horizontal overlaps. When compared to the T-Recs table structure recognition system using the widely-used Marmot dataset [6], this study makes several important contributions to the field:

1. We propose the TEM model, which is a new and advanced deep multi-task architecture specifically developed for detecting tables and columns, as well as recognizing structures. It has shown exceptional performance on the Marmot benchmark datasets [6], setting a new standard in the field;
2. The proposed work demonstrates the efficacy of transfer learning through the process of fine-tuning a pre-trained TEM model using a novel dataset, leading to improved performance of the model.

This paper is organized into different sections. The literature review is discussed in Section 2. The methodology is covered in Section 3, including the details about the proposed classification model. Section 4 of the paper delves into the assessment measures employed, as well as the process of benchmarking and evaluating the algorithm under consideration. Section 5 comprehensively discussed benchmark work and evaluations of the algorithm under consideration. Section 6 further discusses the future work and possible applications. The conclusion is presented in Section 7.

## 2. Literature Review

The deep learning strategy for table recognition in document images was proposed by Schreiber et al. [1]. Their proposed system employs a semantic segmentation model that is based on FCN-Xs architectures. This model incorporates tailored modifications to hyper-features and skip pooling techniques in order to enhance the accuracy of segmentation. Nevertheless, a notable constraint of this approach is the manner in which FCN handles tables. The stride of the FCN filter converts input image pixels into output pixels. However, it fails to consider the distinct recurring patterns found in row–column sequences commonly found in tables. These patterns involve precise constraints for spacing and data length across rows and columns.

The recent studies have extensively explored how to identify and extract tables from documents. Researchers have focused on detecting table structures using heuristic-based methods and deep learning techniques. For instance, Kieninger et al. [2] developed a system called T-Recs, which was one of the early successful attempts to tackle table extraction [2,3,4]. T-Recs works by using word bounding boxes as input, organizing them into rows and columns based on their vertical and horizontal overlaps. However, this method heavily relies on heuristic factors, leading to potential variations in results. Additionally, if the Optical Character Recognition (OCR) stage fails to accurately detect word boundaries, especially for numerical data with missing punctuation like dots and commas, the efficiency of the algorithm can be compromised.

Prior to the deep learning popularity, table detection primarily relied on heuristics or metadata. For example, TINTIN [7] utilized structural clues to identify tables and their components. Another approach involved using hierarchical representations like the MXY tree to detect tables [8], marking the initial use of machine learning methods in this area. Additionally, T. Kasar et al. [9] conducted a study where they employed Support Vector Machine (SVM) classifiers to differentiate between areas that contain tables. Their focus was on identifying intersecting horizontal and vertical lines, along with other low-level characteristics.

Table detection methods have also incorporated probabilistic graphical models. In a study by Silva et al. [10], a Hidden Markov Model (HMM) was utilized to model the joint probability distribution between successive observations of visual page elements and the hidden state of a line. This approach effectively integrated potential table lines into tables, resulting in a considerable level of inclusiveness. Jing Fang et al. [11] detected the table area and decomposed its elements starting from the table header. Conversely, Raskovic et al. [12] focused on identifying borderless tables, using whitespace as a heuristic for detection rather than relying solely on text.

Wang et al. [13] introduced a data-driven approach inspired by the X–Y cut algorithm. This strategy employed probability optimization techniques to address table structure extraction challenges. By considering probabilities derived from a vast training corpus and considering the distances between related terms, this statistical method efficiently handles three column layouts: single column, double column, and mixed column. Shigarov et al. [14] proposed an approach that considers metadata found in PDF files, which contains details about fonts and text bounding boxes. Their system employs ad hoc heuristic principles to recover table cells from a part of text and ruling lines. After integrating these text parts into text blocks using a text block recovery method, the algorithm then applies a threshold to organize the blocks either vertically or horizontally.

Recent developments have led to the introduction of DeepDeSRT [1], a system that employs deep learning strategies for the purpose of table identification as well as table structure recognition. Among these are the determination of the rows, columns, and cell locations within the tables that have been discovered. On the dataset used for the ICDAR 2013 table competition, DeepDeSRT attained performance that is now considered state-of-the-art. Immediately after this, [15] utilized a combination of deep convolutional neural networks, graphical models, and saliency ideas in order to locate tables and charts inside documented papers. An enhanced version of the dataset used for the ICDAR 2013 table competition was used to evaluate this strategy, and the results showed that it performed significantly better than other models that were already developed. It is also important to note that [16] concentrates on finding text components and extracting text chunks. As a consequence of this, the height of every text block is compared to the average height, and, if it satisfies certain requirements, the Region of Interest (ROI) is taken into consideration as a table.

By grouping word segments and assessing text overlap inside the table, T-Recs [4] is one of the early attempts to extract tabular data. It was developed by Y. Wang and colleagues [13] using geometric measurements acquired from a variety of entities contained inside a document. Through the utilization of formatting signals that are available in semi-structured HTML tables, Ashwin et al. [17] are able to extract data from web pages. The process of extracting data from HTML tables is made easier by the fact that tags already demarcate separate cells. The object identification techniques are utilized by Singh et al. [18] in order to gain a knowledge of page layout.

An investigation of table recognition systems was carried out by Zainibbi et al. [19], with the primary focus being on the interactions that occur between table models, observations, transformations, and inferences. The results of their survey give insights into the datasets that are utilized for training and assessment, as well as answers to queries concerning decision-making processes in table structure recognition systems.

A generic technique was given by Jianying et al. [20] for the purpose of extracting table structures from table areas that have previously been recognized. The hierarchical clustering is used in their approach for column detection, and lexical and spatial criteria are utilized for the classification of table headers using this methodology. Utilizing a directed acyclic attribute graph (DAG) is the method that is utilized in order to evaluate the extraction of table structure.

## 3. Methodology

The proposed work is dissected into two parts. At first, we are delineating the process for table detection; later, we explain about the process followed for table extraction.

### 3.1. TEM: VGG-19 for Table and Column Detection

In the earlier development of deep learning methods, table and column detection were viewed as separate challenges. However, when all columns within a document are predetermined, identifying the table region becomes simpler. The columns are of vertical arrangements of words or numbers, and can be difficult to detect individually and may lead to false positives. Understanding the layout of the tabular region can significantly enhance column detection accuracy, as tables and columns often share common regions. Therefore, integrating column-detecting filters into convolutional filters used for table recognition is expected to enhance overall model performance. Our proposed approach draws from the encoder–decoder model for semantic segmentation developed by Long et al. [21] and builds upon this foundational concept.

The encoder of the model is applicable to both table and column detection tasks; however, the decoder is divided into distinct paths for tables and columns. When performing training, the encoding layers are trained using ground-truth data for both tables and columns. Nevertheless, the decoding layers exhibit unique characteristics when applied to the table and column, leading to the creation of two independent computational graphs throughout the training process.

The provided input image undergoes a conversion process to an RGB (Red, Green, Blue) image format, followed by resizing to a resolution of 1024 × 1024 pixels. The altered image is subjected to Tesseract OCR [22]. The generation of output masks for both table and column areas by a single model results in binary target pixel values. These values indicate whether a pixel region belongs to the table/column or background.

Detecting tables in text documents is akin to identifying items in real-world photographs. Similar to generic object detection, table detection relies on recognizing visual characteristics specific to tables. Unlike tasks involving object identification, table and column detection require a lower tolerance for noise. Our approach involves employing pixel-wise prediction to identify table and column areas, rather than solely predicting their borders. Recent advancements in semantic segmentation have highlighted the effectiveness of encoder–decoder network architectures, such as the Fully Convolutional Network (FCN) developed by Long et al. [21,23]. FCN architecture utilizes skip connections to merge low-resolution decoder feature maps with high-resolution encoder features, with VGG-19 serving as the base layer. Fractionally stridden convolution layers are then used to upscale the low-resolution semantic map, with additional high-resolution encoding layers integrated into the upsampled map. Fractionally stridden convolutional layers, also known as Transposed Convolution, are operations used in deep learning and convolutional neural networks (CNNs) for upsampling feature maps.

**Figure 1:**
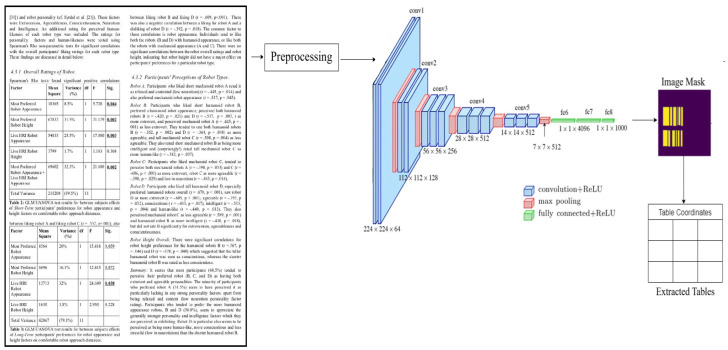
Graphical Representation of TEM Architecture.

The method employed in our model is analogous to that of the encoder/decoder network observed in the FCN [21] design. The suggested model, as seen in Figure 1, employs a pre-trained VGG-19 layer as the foundational network. The VGG-19 model replaces its fully linked layers (layers after pool5) with two (1 × 1) convolution layers. In Figure 1, it can be observed that each convolution layer (conv6) is succeeded by a Rectified Linear Unit (ReLU) activation and a dropout layer with a dropout probability of 0.8 (conv6 + dropout). Following this, two distinct subsections of the decoder network are included. The underlying principle of this design is predicated on the notion that the column area is a subset of the table region. Hence, a solitary encoding network possesses the capability to effectively eliminate active areas by using attributes derived from both table and column regions. Convert the input image to grayscale by empirically selected values and apply a threshold to create a binary mask.

(1) $$GrayScaleImage\left(I_{g}\right)=0.2989.I_{r}+0.5870.I_{g}+0.1140.I_{b}$$

After thresholding,

$$I_{th}\left(x,y\right)=\left\{\begin{array}{c} 0\;\;\;\;if\;\;I_{g}\left(x,y\right)<T\\ 1\;\;\;\;if\;\;I_{g}\left(x,y\right)\geq T \end{array}\right.$$

where

$I_{r}$, $I_{g}$, $I_{b}$ are the red, green, and blue components of the original image, respectively.

*T* is the threshold value for binarization. Use morphological operations to detect horizontal and vertical lines corresponding to table rows and columns. Apply morphological operations (erosion followed by dilation) using a rectangular kernel that is wider than it is tall; mathematically, this relation is defined in Equation (2):

(2) $$I_{h}\left(x,y\right)=dilate\left(erode\left(I_{th},K_{h}\right),K_{h}\right)$$

where $K_{v}$ is a kernel for detecting vertical lines (e.g., a rectangle of size h × 1, where h is the height of the expected vertical line). Combine horizontal and vertical masks to isolate the table structure:

(3) $$I_{mask}\left(x,y\right)=I_{h}\left(x,y\right)\cap I_{v}\left(x,y\right)$$

This mask defines the regions corresponding to the table grid. For each detected vertical line in $I_{v}\left(x,y\right)$, calculate the bounding boxes that define individual table columns. Assume that the *i*-th vertical line is located at $x_{i}$, as explained mathematically in Equation (4):

(4) $$Bounding\;Box\;for\;Column\;i=(x_{i},x_{i+1},y_{min},y_{max})$$

where $x_{i}$ and $x_{i+1}$ are consecutive vertical lines (column separator), and $y_{min},y_{max}$ are the top and bottom of the table (as detected from $I_{h}$).

Both decoder branches obtain the output of the (conv6 + dropout) layer. The table branch utilizes an extra (1 × 1) convolution layer called conv7 table, which is then followed by a sequence of fractionally stridden convolution layers to increase the resolution of the image. In addition to being upscaled using fractionally stridden convolutions, the output of the conv7 table layer is additionally mixed with the pool4 pooling layer of the same dimension. Similarly, the feature map that has been merged is subjected to further upscaling and then combined with the pool3 pooling layer of the same dimension. This is followed by upscaled scaling to align with the original image dimension.

The column detection branch incorporates an additional convolution layer (conv7 column) that use a Rectified Linear Unit (ReLU) activation function. Additionally, a dropout layer is incorporated, with the same dropout probability. After (1 × 1) convolution (conv8 column) layer, the feature maps are upsampled using fractionally stridden convolutions. The feature maps that have been upsampled are combined with the pool4 pooling layer. Subsequently, the resulting combined feature map is further upsampled and mixed with the pool3 pooling layer of the same dimension, until the original image size is achieved. Prior to the transposed layers, both branches utilize numerous (1 × 1) convolution layers. The utilization of (1 × 1) convolution is intended to decrease the size of feature maps (channels), which is essential for the prediction of pixel class. The channels of the output layers, also known as the encoder network output, should be equal to the number of classes. In this case, the channel with the highest probability is allocated to the relevant pixels. As a result, the computational graphs’ outputs provide masks for the table and column areas.

### 3.2. Table Data Extraction

**Figure 2:**
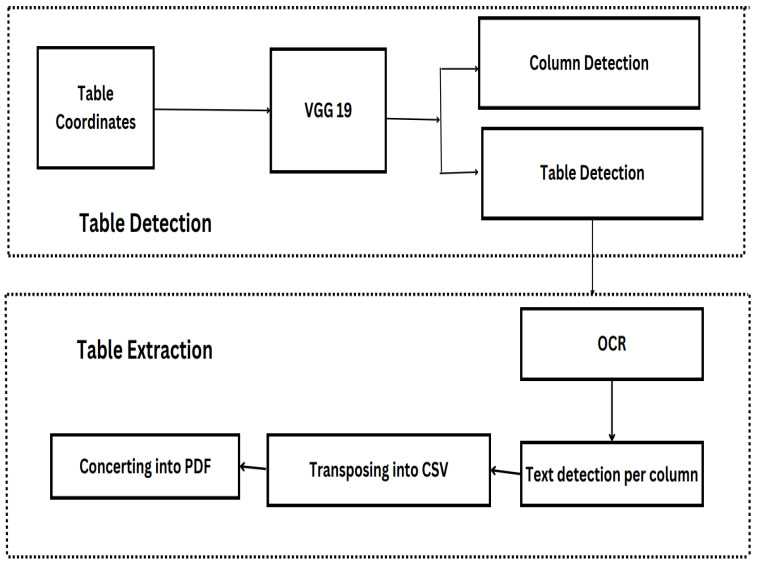
TEM Architecture Flow Chart.

The proposed table detection and content extraction model (TEM) is represented in Figure 2. Using TEM for document processing, masks depicting the table and column areas are produced. The primary purpose of these masks is to effectively separate the table and its column sections from the overall image. Given the pre-existing knowledge of word placements in the document, as determined by Tesseract OCR, only word patches situated inside the table and column areas are retrieved. Within each detected column, apply an OCR (Optical Character Recognition) algorithm (such as Tesseract) to extract text, as represented mathematically in Equation (5).

(5) $$Text\left(i\right)=OCR(I\left(x_{i}:x_{i+1},y_{min}:y_{max}\right))$$

A row can be defined as a group of words from many columns that are at a comparable horizontal level, using the filtered words. It is noteworthy that a row is not inherently limited to a solitary line; rather, the extent to which a row extends over many lines is contingent upon the content of a column or the demarcations of the line. Consequently, in order to tackle these differences, we create three guidelines for dividing rows:

- In tables where line demarcations are visible, it is common for these lines to separate rows inside each column. In order to identify possible line boundaries for rows, we analyze each gap between two words that are vertically aligned in a column. This is carried out by applying a Radon transform [24] to see if there are any horizontal lines present. Horizontal line delineation facilitates the distinct separation of rows;
- In the case when a row extends across many lines, the initial position for a new row is determined by selecting the rows that contain the highest number of non-blank entries. In the context of a multi-column table, it is possible for certain columns to consist of items that span a single line, such as amount, while others may encompass data that span many lines, such as description. Consequently, every subsequent row begins after all columns have been populated with entries;
- In instances where all columns are completely populated and there are no line demarcations, it is possible to regard each line or level as an individual row.

### 3.3. Dataset Preparation

Deep learning methodologies are strongly dependent on data and often need substantial quantities of training data in order to acquire proficient representations. Nevertheless, there is a dearth of datasets that are explicitly tailored for the purpose of table identification, such as the Marmot dataset [6], which has about 1000 table images. Furthermore, there is a scarcity of datasets specifically designed for the purpose of identifying table structures. The only effort is by [6], which proposed the Marmot_data table competition dataset. The dataset contains images and xml files with the coordinates of tables existing in the images. The limited availability of data is a significant obstacle for deep learning models that seek to address the tasks of table recognition and table structural analysis. In order to train our model, we employed the Marmot table recognition dataset, which was divided into a training set and a testing set, with a ratio of 90/10. Additionally, we normalized the value to fine-tune the model by dividing it to 255. Then, decode the image through a tensor flow decoder for mask recognition.

### 3.4. Image Pre-Processing

Preprocessing table images to reduce their structure and make the table arrangement more visible is the first step in processing these images. Improving the classifier’s efficiency by removing extraneous image information relies heavily on this transformation. The first step is image cleaning, which entails erasing any foreground components other than text, such as ruling lines. After that, adaptive binarization is used on the cleaned images to make sure that the pixel intensities are consistent everywhere. The images are reduced in size to 1600 × 512 after binarization, because this is the input size that the neural network is specifically designed to process. The images are subjected to three rounds of dilation alteration using rectangular kernels after resizing. To identify columns, a vertical dilation filter with dimensions of 3 × 5 is utilized, whereas a horizontal dilation filter with dimensions of 5 × 3 is used for row recognition. The model is able to learn the patterns of row and column separators more successfully since these dilation operations connect nearby rows and columns. In order to make the changed images ready to be fed into the next recurrent neural network, they are normalized to a range of 0 to 1.

### 3.5. Providing Semantics Information

Tables inherently possess data types that are constant within consecutive rows or columns, contingent upon whether the table adheres to a row-major or column-major structure. As an illustration, a column denoted as “Name” commonly comprises textual data, but a column denoted as “Quantity” has numerical data. The semantic information is included into the deep learning model by color-coding text sections that have comparable data kinds. The network utilizes the color-coded representation of the text sections as input, leading to enhanced model performance. In addition, spatial semantic aspects have been included by emphasizing word patches according to their respective data kinds. After preprocessing, Tesseract OCR [22] is used to extract word blocks. The word patches are subsequently assigned colors based on their fundamental data type. The network receives the changed images with color-coded word patches as input. The TEM architecture is designed to analyze the input image and produce binary mask images for individual tables and columns. The result produced is subsequently enhanced by the use of predetermined rules that are derived from the identified table and column masks. An illustrative sample of the produced output is presented.

## 4. Evaluation

The initial step involves the utilization of Tesseract OCR to process the image, whereas all word patches are extracted from the document image. Subsequently, regular expressions are employed to ascertain the data type of these words. The underlying justification for employing color-coding in word bounding boxes is to effectively communicate both semantic and spatial information to the neural network. A distinct color is allocated to each data type, and word bounding boxes with comparable data kinds are given the same colors. This procedure facilitates the elimination of false detection within the word bounding boxes. In order to properly manage scenarios when noise is introduced during word identification and extraction from OCR, it is necessary to train the model accordingly. In order to replicate the process of partial word identification during training, a small number of word patches are intentionally excluded at random. This procedure facilitates the acquisition of resistance in the model towards missing or noisy data. In contrast to the original document image, the color-coded image exhibits a deficiency in visual elements such as line demarcations, corners, and color highlights, despite its use for training purposes. In order to preserve these significant visual characteristics in the training data, the image with highlighted words is merged with the original image on a pixel-by-pixel basis. The model is trained using modified document images that contain both semantic information and original visual attributes.

During the training of our models, we utilized the Adam optimizer in conjunction with the binary cross-entropy loss function. Table images often consist of a greater number of rows and columns compared to the empty spaces between them. A class imbalance issue was seen during the early training efforts, when the model consistently made predictions for row–column items but faced difficulties in detecting white-space regions. In order to rectify this issue of imbalance, we implemented weighting into our loss function. The weighting technique employed in this study resulted in a penalty of just 66% for erroneously predicted row–column components compared to mistakenly predicted white-space elements, thereby achieving a balanced learning process.

## 5. Experiment and Results

Page segmentation frequently employs two standard measures, namely, precision and recall. When an area encompasses all the sub-objects that are present in the ground-truth, it is deemed to be complete. The sub-objects refer to the smaller elements or components within a table cell that are relevant for text recognition and extraction. An area is classified as pure when the elements within that area align perfectly with what is expected based on the manually annotated ground-truth data. Subordinate entities are commonly established by partitioning the provided area into significant components, such as table heads, table contents, and so forth. The assessment process considers individual characters inside each zone as sub-objects. Subsequently, precision and recall metrics are generated by considering the sub-objects present inside each region. The average values are then computed for all regions within a particular text.

The adjacency connections are the closest horizontal and vertical neighbors. The adjacency relations are denoted as a one-dimensional tuple that encompasses the textual data of the adjacent cells. In order to enhance the comparability with established data, the content within each cell underwent normalization procedures. These procedures involved the removal of white spaces, substitution of special characters with underscores, and conversion of lowercase letters to uppercase. TEM cannot learn about structures without annotated data, which are necessary for both table identification and training. The dataset includes 1016 images with tables in both Chinese and English. Out of them, 504 images in English have been annotated and utilized for training. This deep learning model was built using Tensorflow and executed on a machine that had an Intel(R) Core(TM) i5-8265U CPU @ 1.60 GHz, 1.80 GHz, 8 GB of RAM, 64-bit operating system.

### Experiments

**Figure 3:**
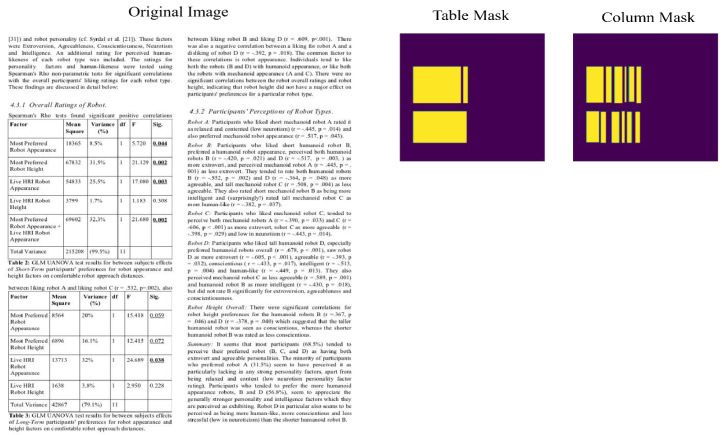
Table Mask and Column Mask.

The first experiment tested the model’s ability to recognize structures and tables on the Marmot_data table competition dataset after training it with all positive samples from the Marmot dataset. A computation graph for the table region and another for the column region were used in the training procedure. A document image, a table mask, and a column mask make up each training sample. There was a 2:1 ratio of table region to column region calculations during the first training period. Prior to its specialization in recognizing column areas, the model was trained to produce enormous active tabular regions. With a learning rate of 0.0001 and a batch size of 2, the training was adjusted to a 1:1 ratio for both branches until convergence after around 25 iterations. We used a validation set from the Marmot_data dataset to keep an eye on convergence and overfitting, while we kept training for 25 iterations with the same training pattern and optimizer parameters. In order to reliably identify the table and column sections, pixel-wise prediction was employed during testing with a threshold probability of 0.99. Hence, we can then obtain a column mask and table mask using different batch sizes with an improved accuracy of 87%, where an image from test dataset is passed to the trained model and a table mask is generated. Subsequently, the column mask is generated, as shown in Figure 3.

**Figure 4:**
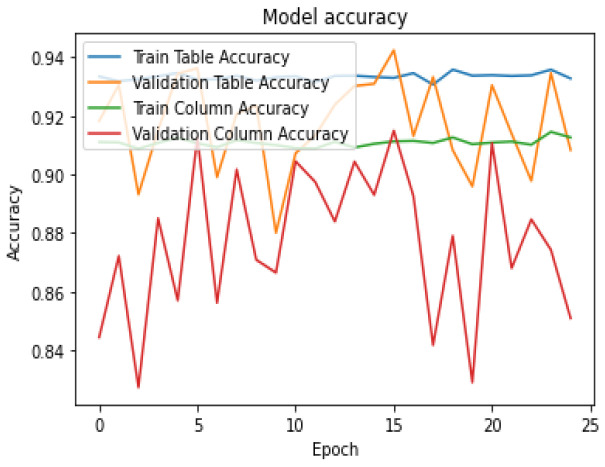
Model Accuracy during Training.

**Figure 5:**
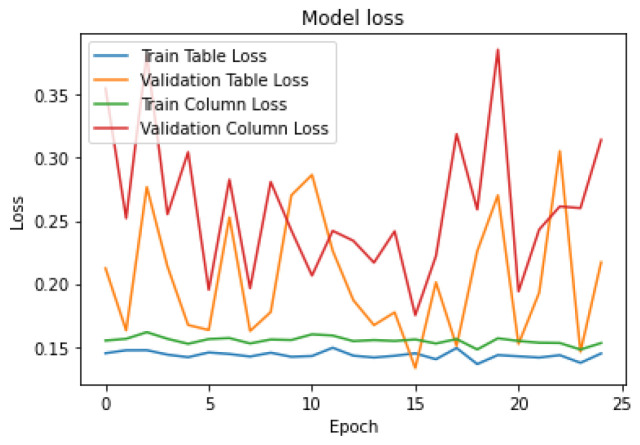
Loss Representation of Table Mask and Column Mask.

To evaluate how well our model is learning from the data during training, we generated two types of curves: model accuracy and model loss. These curves show us how accurate our model’s predictions are and how much error or loss it experiences over time. We trained the model for 25 epochs, which means we ran the training process for 25 iterations or until it passed through the entire dataset. These curves can be seen in Figure 4 and Figure 5.

Our study compared various methods for image and PDF processing in terms of accuracy. Table 1 summarizes the results, showcasing the performance of different methods, including Hsu et al. [25], Yildiz et al. [26], Khan, Saqib Ali et al. [27], and our proposed method TEM. Our approach demonstrated superior accuracy compared to existing methods, particularly in handling image inputs, achieving an accuracy of 0.876.

## 6. Discussion and Future Work

Table detection and extraction are key steps in processing structured information from documents. These tasks become challenging when documents are noisy or complex. Noise, such as blurred scans, faded text, or random marks, makes it difficult to clearly identify table boundaries. Similarly, documents with intricate layouts, such as those with nested tables, uneven rows, or mixed alignments, can confuse traditional models. These issues are common in scanned files, older documents, or those created with inconsistent formatting, making table extraction a tricky process.

Our model addresses these challenges using VGG-19, a deep learning model designed to analyze visual patterns. It learns to recognize table structures even when the documents are noisy or their layouts are complex. By incorporating semantic information, the model not only detects tables, but also accurately identifies column boundaries, which is critical to extracting meaningful data. This capability ensures that the model performs well even in cluttered or irregular scenarios, providing reliable results where other methods may fail.

This research has significant practical applications. In finance, for example, the model can help automate the extraction of tables from reports, invoices, or tax documents, saving hours of manual effort. For medical records, it can organize patient data stored in tabular formats, making it easier for healthcare professionals to access critical information quickly. These applications not only save time but also minimize errors, improving efficiency in industries that rely on accurate data processing.

In responding to real-world challenges, the research highlights the importance of improving table detection and extraction methods. The model’s ability to handle noisy and complex documents makes it a valuable tool for automating document processing. This can benefit industries such as healthcare, finance, and education, where structured information often needs to be extracted from diverse and complex documents. The work demonstrates how advanced AI methods can solve everyday problems, making processes faster, more accurate, and cost-effective.

## 7. Conclusions

This study presents TEM, a revolutionary deep learning model that has been trained to tackle the end-to-end tasks in structure identification and table detection simultaneously. The interesting thing about TEM is that it tackles both problems at once, unlike other approaches that perform each in isolation. By utilizing the fundamental link between table detection and structure identification, TEM pioneers a unified strategy to solve these problems that exist in table structure and data manipulation. In this research, we provide a new approach to deep learning table structure extraction using GRU-based sequential models. Due to sequence models’ high representation capabilities, which effectively capture the recurrent row/column patterns inherent in tables, this technique exhibits substantial improvement compared to heuristic approaches and DNN-based models. In the future, we want to build a complete framework for data extraction from table cells.

## Figures and Tables

**Table 1:** Comparison Table.

Study	Method	Input Type	Accuracy
ICDAR table comparison	Hsu et al. [25]	PDF	0.5220
Extract table information from PDF files	Yildiz [26]	PDF	0.7313
Table extraction using GRU	Khan, Saqib Ali, et al. [27]	Images	0.55
Table detection and extraction with labels	TEM (Proposed)	Images	0.876

## Data Availability

Data are contained within the article.
